# Atypical presentation and transabdominal treatment of chylothorax complicating esophagectomy for cancer

**DOI:** 10.1186/1749-8090-7-9

**Published:** 2012-01-24

**Authors:** Matteo Rottoli, Iris S Russo, Daniele Bernardi, Luigi Bonavina

**Affiliations:** 1 University of Milano, Department of Surgery, IRCCS Policlinico San Donato, Piazza E. Malan 2, San Donato Milanese (Milano), Italy

**Keywords:** Esophagectomy, thoracic duct, cisterna chyli, chylotorax, cardiac tamponade, transhiatal approach

## Abstract

Chylotorax is a relatively uncommon and difficult to treat complication after esophagectomy for cancer. We report a case of a young adult male who underwent neoadjuvant chemoradiationtherapy followed by Ivor-Lewis esophagectomy for a squamous-cell carcinoma of the distal esophagus. During the postoperative course the patient presented recurrent episodes of hemodynamic instability mimicking cardiac tamponade, secondary to compression of the left pulmonary vein and the left atrium by a mediastinal chylocele. Mediastinal drainage and ligation of the cisterna chyli and the thoracic duct was successfully performed through a transhiatal approach.

## Introduction

Chylotorax is a rare but potentially life-threatening complication of esophagectomy [1]. It occurs mostly after transthoracic esophagectomy and is often associated to pulmonary complications [2]. The daily amount and duration of chyle drainage dictate the need and timing for reoperative surgery. An initial trial of conservative treatment is often justified because it allows undisturbed healing of the esophagogastric anastomosis [3]. On the other hand, postponing a reoperation too late may lead to severe metabolic, immunologic, nutritional, and cardiorespiratory consequences. We report the case of a patient with postoperative chylotorax who presented with hemodynamic instability mimicking cardiac tamponade and was treated through a transhiatal approach after an unsuccessful attempt at conservative management.

## Case presentation

A 48 year old man was referred to our center with a 5 month history of dysphagia to solid food and weight loss. There was no significant comorbidity. The esophagogastroduodenoscopy showed an infiltrating tumor of the lower esophagus located between 33 and 38 cm from the incisors. Histopathologic examination revealed a poorly differentiated squamous cell carcinoma. Chest and abdominal computed tomography scan confirmed the presence of the esophageal lesion with enlarged mediastinal nodes and no evidence of distant metastases. The patient underwent neoadjuvant chemotherapy with carboplatin, paclitaxel, 5-fluorouracil, and 46 Gy radiotherapy. The re-staging after neoadjuvant treatment showed a partial response. The patient underwent a Ivor-Lewis esophagectomy with 2-field lymphadenectomy via laparoscopy and right thoracotomy. As usual, the thoracic duct was ligated *en bloc *with the azyos vein above the diaphragm. Pathological examination of the surgical specimen showed yT2-N1 disease and no evidence of margin infiltration.

**Figure 1:**
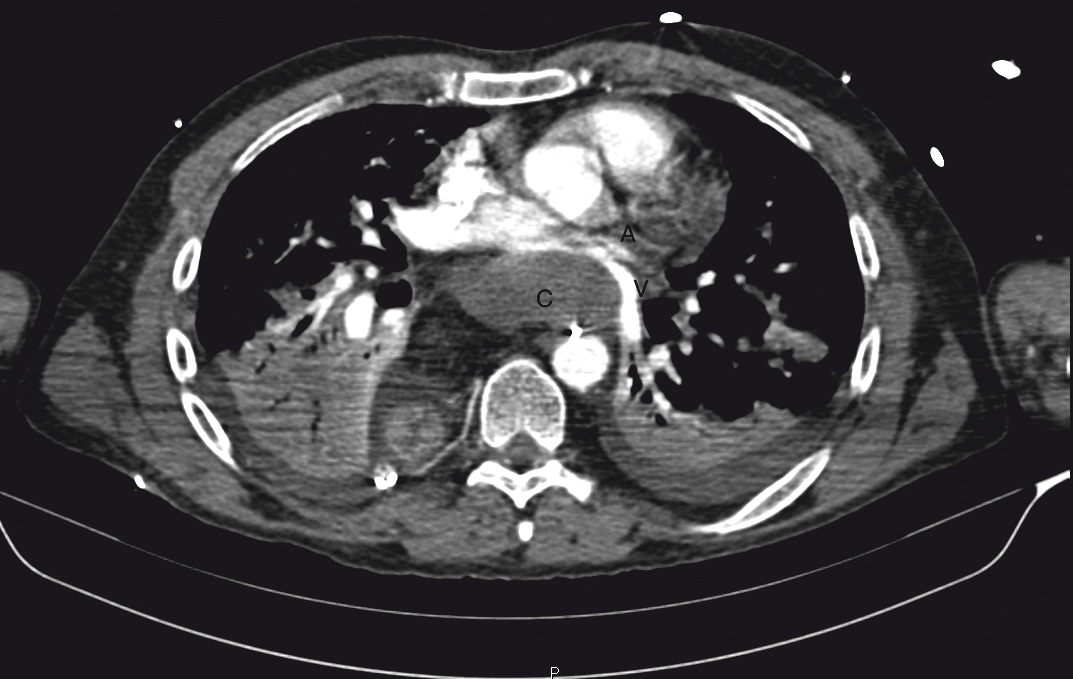
**Retrocardiac fluid collection causing compression of the left pulmonary vein and the left atrium**. C: fluid collection. A: left atrium. V: left pulmonary vein.

A high-volume serous fluid output (1100-1700 ml/day) from the right chest drain was noted since postoperative day 1. On postoperatively day 3 a left pleural effusion was detected on the chest x-ray and about 2 liters of serous fluid were drained. Biochemical analysis of triglyceride contents from both drains confirmed the diagnosis of chylothorax. The subsequent hospital course was characterized by recurrent episodes of dyspnea with supraventricular tachycardia and systolic hypertension refractory to pharmacological therapy. A gastrographin swallow study was performed on postoperative day 10 and was normal. The patient was then allowed a semiliquid medium-chain triglyceride diet, but the drain output changed from yellowish to milky and the chyle drainage continued despite treatment with continuous intravenous infusion of etilefrine and subcutaneous octreotide. On postoperative day 22 another major episode of acute respiratory distress with hemodynamic instability occurred. A CT scan demostrated the presence of a retrocardiac fluid collection, extending from the level of the esophagogastric anastomosis to the diaphragm and causing compression of the left pulmonary vein and the left atrium (FIGURE 1). This finding was confirmed by a transthoracic echocardiogram.

The patient was then taken to the operating room. An esophagogastroduodenoscopy performed under general anesthesia showed a 1 cm defect on the left lateral wall of the stapled esophagogastric anastomosis without evidence of ischemia of the gastric conduit. Therefore, a 28 mm covered Ultraflex stent (*Boston Scientific*) was delivered under fluoroscopic control. An upper midline laparotomy was then performed. After gentle displacement of the gastric tube, a loculated collection containing milky fluid was digitally ruptured and drained. In addition, transhiatal multiple ligations of the *cisterna chyli *and the thoracic duct were performed on the right side of the aorta. A feeding jejunostomy was also performed. Drainage of chyle was no longer observed in the mediastinal and chest tubes, and all signs of hemodynamic instability disappeared after the reoperation.

The postoperative course was complicated by thrombosis of the left brachiocephalic vein requiring continuous heparin vein infusion that was mantained until the 32nd postoperative day, when complete resolution of the thrombosis was demostrated by CT scan and ultrasonography. The subsequent hospital course was further complicated by sepsis from *Pseudomonas Aeruginosa *and *Acinetobacter Baumanii *requiring antibiotic therapy with Gentamicin and Piperacillin-Tazobactam for 15 days until complete resolution of fever and bacteremia. The patient was discharged in good general conditions and eating a regular diet on the 54th postoperative day.

### Comment

The diagnosis of chylothorax complicating a Ivor Lewis esophagectomy was made early in the postoperative course of this patient. Conservative treatment with etilefrine and octreotide was unsuccessful, and despite the presence of bilateral chest drains, a posterior mediastinal collection containing chyle developed that caused an atypical clinical picture with recurrent episodes of hemodynamic instability. Supraventricular tachycardia and systolic hypertension mimicking cardiac tamponade were likely to be caused by extrinsic compression of the left pulmonary vein and atrium as documented by CT scan and echocardiogram, and were refractory to pharmacologic therapy. Similarly, Barbetakis et al reported on a young female who was admitted with a true cardiac tamponade due to a large chylous pericardial effusion and, after the periocardiocentesis, was successfully treated by a pericardial window and ligation of the thoracic duct through a right minithoracotomy [4].

The standard of care in patients with post-esophagectomy chylothorax is the trans-thoracic supradiahragmatic ligation of the thoracic duct. The operation can be carried out through a right thoracotomy or a thoracoscopic approach [5]. In our patient, due to the endoscopic evidence of an esophagogastric anastomotic leak, we preferred a transhiatal rather than a transthoracic approach because of the fear to create further damage to the anastomosis. The transabdominal approach to the cisterna chyli and thoracic duct, already reported in the literature [6,7], was particularly appropriate in this patient because it allowed both the drainage of the loculated mediastinal collection and the ligation of the thoracic duct at the origin from the cisterna chyli.

### Consent

Written informed consent was obtained from the patient for publication of this Case report and the accompanying image. A copy of the written consent is available for review by the Editor-in-Chief of this journal.

## Competing interests

The authors declare that they have no competing interests.

## Authors' contributions

MR: conception and design, analysis and interpretation of data, drafting of the manuscript, critical revision, final approval; ISR: data collection, design, analysis of data, drafting of the manuscript, final approval; DB: data collection, design, analysis of data, drafting of the manuscript, final approval; LB: conception and design, interpretation of data, drafting of the manuscript, critical revision, final approval.
